# Cataract Surgery to Lower Intraocular Pressure

**DOI:** 10.4103/0974-9233.56222

**Published:** 2009

**Authors:** John P. Berdahl

**Affiliations:** From Minnesota Eye Consultants, 710 East 24^th^ St, Suite 100, Minneapolis MN 55404-3810

**Keywords:** Cataract, Surgery, Phacoemulsification, Glaucoma, Intraocular Pressure

## Abstract

Cataract and glaucoma are common co morbidities. Cataract surgery is frequently performed in patients with glaucoma. In this study, a review of literature with search terms of cataract, glaucoma and intraocular pressure is followed by evaluation and synthesis of data to determine the effect of cataract surgery on intraocular pressure. Cataract surgery seems to lower intraocular pressure on a sustained basis, especially in patients with higher preoperative intraocular pressure. The mechanism of action of these finds remains speculative.

## INTRODUCTION

Cataract and glaucoma are the first and second leading causes of blindness worldwide.^1^,^2^ Although usually not severe enough to cause blindness, it is not surprising that these two diseases occur simultaneously in many patients. Many studies have demonstrated intraocular pressure reduction after cataract surgery.^3^ However, most recent data indicates that IOP reduction after cataract surgery is more significant and sustained than previously thought.^4^

The mainstay of glaucoma treatment is to lower intraocular pressure. Traditional glaucoma surgeries such a trabeculectomy and tube shunts work well to lower intraocular pressure and decrease progression of glaucoma, but these procedures carry significant risk.^5^,^6^ Many patients with glaucoma have concurrent cataracts and some studies have suggested that glaucoma itself is a risk factor for cataract development.^7^–^9^ Glaucoma filtering procedures, peripheral iridotomy and some glaucoma medications increase the risk of cataract formation.^5^,^10^–^17^ Historically, patients with moderate to advanced glaucoma with concurrent cataracts would have either a combined procedure or a two-stage surgery.^6^,^18^–^22^ Surgeons have traditionally felt that cataract surgery lowers IOP in open angle glaucoma (OAG) only slightly and temporarily - despite a paucity of robust data.^23^ In contrast, current data demonstrates a greater and more sustained IOP reduction.^4^,^10^ As such, cataract surgery may be a safe alternative to glaucoma surgery in some patients and could shift the surgeon's approach in treatment of concurrent cataract and glaucoma.

## SUMMARY OF STUDIES

The earliest studies of intraocular pressure after cataract surgery showed little if any reduction of intraocular pressure.^24^–^26^ However, these results probably don't apply today because of advances in surgical technique and intraocular lens technology. As extra capsular surgery became the standard, studies began to demonstrate a lowering of IOP^27^ sometimes on a sustained basis.^10^ The conventional wisdom that cataract surgery lowers IOP by two to four— mmHg for a couple years was partially confirmed by the only meta-analysis of the topic.^23^ This meta-analysis showed a long term mean IOP reduction of two to four— mmHg. However, long term was defined as longer than 24-hour follow-up (although many studies had even longer follow-up). Additionally, data in nearly all of the studies showed only mean IOP change and did not stratify patients based on preoperative IOP which has critical significance when interpreting the data. Studies which did stratify patients based on preoperative IOP clearly demonstrated that patients with higher preoperative IOP enjoy the greatest reduction of IOP after cataract surgery.^4^,^28^,^29^ Longer term studies have shown a drop in IOP of about three mmHG in POAG patients and nonglaucoma patients with 75-85% of patients maintaining a IOP reduction at five years.^30^–^34^ IOP can be controlled in 20% of patients with OAG without drops following cataract surgery.^35^

The method of cataract extraction may influence the reduction of IOP. Phaco emulsification (particularly clear cornea phaco emulsification) seems to lower IOP more than manual extra capsular cataract extraction.^10^,^27^,^36^,^37^ The type of OAG may also influence IOP reduction. Pseudo exfoliation patients may have an even greater long term decrease in IOP than POAG patients even though IOP often rises on postoperative day one.^38^–^40^ However, early IOP variability following cataract surgery has not been associated with clinical consequence.^41^ Many other factors such as pressurization at the time of surgery, immediate postoperative medications, and visco elastic type contribute to short term IOP fluctuations following surgery.^42^–^44^

### Pathophysiology of Reduced IOP after Cataract Surgery

Although the physiological reasons for decreased IOP after cataract surgery remain speculative, the facility of outflow is known to increase after cataract surgery.^45^ The angle width does not change in normal or OAG patients after cataract surgery suggesting improved function of the trabecular meshwork itself rather than improved aqueous access to the trabecular meshwork.^46^ Three or more different mechanisms may contribute to the observed reduction in IOP after cataract surgery.

### Lens-induced Changes to Outflow Pathway

As the eye ages, the crystalline lens increases significantly in volume. This may initiate a series of anatomical changes that ultimately leads to the increase in IOP observed with aging.^47^,^48^ As the lens grows, the anterior lens capsule is displaced forward causing the zonules to place anteriorly directed traction on the ciliary body and uveal tract, which in turn compresses the canal of Schlemm and the trabecular meshwork. Since the anterior tendons of the ciliary muscles contribute to the architecture of the trabecular meshwork, as the ciliary body is displaced forward by the enlarging lens the tendons relax and the space between trabecular plates becomes narrowed.^49^

### Inflammation Induced by Cataract Surgery

Phaco emulsification typically induces a low grade inflammation in the immediate postoperative period.^50^,^51^ It is plausible that the induced inflammation lowers IOP by either decreasing aqueous production of the ciliary body as seen in uveitis; or it could increase outflow similar to the mechanism of selective laser trabeculoplasty or prostaglandin analogues. Although these options seem plausible, little experimental data exists to support these hypotheses.

### Fluidics of Phaco Emulsification

An additional explanation is that high flow of fluid and high IOP (up to 90mmHg) experienced during cataract surgery forces fluid through the trabecular meshwork into the canal of Schlemm and the episcleral veins.^52^,^53^ Forcing this large amount of fluid through the drainage system may increase patency and promote flow. Again, there is little evidence to support or refute this hypothesis.

### Should cataract surgery be considered a glaucoma surgery?

Cataract surgery is a very common, successful, highly refined surgery with a favorable risk/benefit profile including improved visual acuity and visual field.^54^ The widespread general belief that cataract extraction alone lowers IOP two to four mmHg is slowly evolving towards an understanding of a larger and more sustained IOP reduction, especially in patients with higher preoperative IOP.^4^,^23^,^55^ Even though cataract surgery alone lowers IOP, combined glaucoma/cataract surgery lowers IOP more with fewer postoperative pressure spikes.^23^,^56^ Surgeons should carefully monitor IOP after cataract surgery to prevent a postoperative pressure spike that could “snuff” the nerve especially in patients with pseudo exfoliation syndrome.^41^,^57^,^58^ Cataract surgery to lower IOP may be especially beneficial in developing contries or are where the close follow-up necessitated by traditional glaucoma surgery is difficult. Nonetheless, cataract surgery seems to be emerging as a safe way to lower IOP in patients with mild to moderate glaucoma while avoiding the morbidity of traditional glaucoma surgery.
